# Changes in ion-channels in the dorsal root ganglion after exposure to autologous nucleus pulposus and TNF. A rat experimental study

**DOI:** 10.1016/j.jor.2023.11.012

**Published:** 2023-11-04

**Authors:** Joakim Håkansson, Oskar Juhlin, Armen Hovannisyan, Jennifer Rosendahl, Yalda Bogestål, Kjell Olmarker

**Affiliations:** a RISE Research Institutes of Sweden, Unit of Biological Function, Division Materials and Production, Borås, Sweden; b Department of Laboratory Medicine, Institute of Biomedicine, University of Gothenburg, Gothenburg, Sweden; c Department of Chemistry and Molecular Biology, University of Gothenburg, Gothenburg, Sweden; d Musculoskeletal Research, Department of Medical Chemistry and Cell Biology, Institute of Biomedicine, Sahlgrenska Academy, University of Gothenburg, Gothenburg, Sweden

**Keywords:** Spine, Sciatica, Low back pain, Ion channels, Rats

## Abstract

**Purpose:**

It is known that contact of nucleus pulposus with the dorsal root ganglion may induce changes in nerve conduction and pain behavior. It has also been suggested that the behavioristic changes are caused by changes in voltage-gated ion channels, which in turn have been upregulated by TNF. Such upregulations have previously been shown for NaV 1.8 and NaV 1.9. In this investigation, we expanded the number of studied ion channels after the application of nucleus pulposus or TNF.

**Methods:**

Following removal of the left L4-5 fact joint, a disc puncture was performed and the dorsal root ganglion was exposed to nucleus pulposus (n = 5) and TNF (n = 5). Operated rats without disc puncture served as sham (n = 5) and 5 non-operated (naïve) rats were included. After 24 h, the DRGs were harvested and analyzed by quantitative PCR on validated pre-spotted primer plates displaying genes for 90 voltage-gated ion channels.

**Results:**

It was evident that the changes in operated animals were separate from the naïve rats. It was also apparent that gene expression changes in rats with nucleus pulposus or TNF application showed similar trends and were also separated from sham-operated animals.

**Conclusion:**

The application of nucleus pulposus and TNF onto the DRG in rats induces comparable changes in gene expression of several ion channels. Since the changes induced by TNF and NP are similar, one might also suspect that TNF mediates the NP-induced changes. However, such a mechanism needs further investigation.

## 1 Introduction

During the last years it has become more evident that mechanical deformation of nervous tissue does not necessarily induce pain and that the pain produced, as for instance at nerve root deformation in sciatica, is more likely to be the result of deformation of sensitized nervous tissue.^1^^,^^2^ It is, however, not clear how such sensitization might occur, but it seems likely that substances from the herniated nucleus pulposus may be involved. Tumor Necrosis Factor alpha (TNF) has been suggested to be one such mediator.^3^ In a previous rat study, it was shown that exposure of the dorsal root ganglion to nucleus pulposus induced upregulation of the voltage gated sodium channels (VGSC) NaV 1.8 and NaV 1.9 ^4^. VGSC has a major role in regulating the excitation of neurons, and are located in the plasma membrane of the cell where they mediate influx of ions passing through in response to local membrane depolarization.^5^ A shift in the expression or functionality of VGSC, profoundly alters the excitation pattern of sensory neurons in the dorsal root ganglion (DRG) as well as neurons in the central nervous system. It may therefore cause firing of ectopic signals in the nervous system.^6^^,^^7^ In this investigation, we aimed at expanding the assessment of changes in ion channel density after exposure to nucleus pulposus by assessing a higher number of ion-channels using quantitative PCR. Since TNF has previously been shown to be of interest in sciatic pain production, we also investigated the effects of TNF *per se*.

## 2 Methods

### 2.1 In vivo model

The animal experiments were conducted following approval from the local ethics committee for animal studies in Gothenburg, Sweden, and in compliance with EU Directive 2010/63/EU for animal experiments. Twenty female Sprague–Dawley rats (Charles River, Germany) weighing around 250 g on average were utilized. The rats underwent a minimum one-week acclimatization period before the surgery and were group-housed with three to four rats per cage and had unrestricted access to food and water. Anesthesia was induced through inhalation of isoflurane (Isobavet, Schering-Plough Animal Health, Farum, Denmark), and pain relief was administered via subcutaneous injections of buprenorphine (48 μg/kg, Temgesic, Schering-Plough, Brussels, Belgium). To expose the left L4 dorsal root ganglion, the left facet joint between vertebrae L4 and L5 was excised following a midline incision of the skin and the thoracolumbar fascia. In the sham group (n = 5), the wound was closed at this point and no further procedure was performed on the DRG. The spinal muscles were sutured and the skin was closed with metal-clips. Within the NP group (n = 5), the L4-5 disc adjacent to the DRG was punctured using a 0.4 mm diameter needle. Subsequently, 0.2 ml of air was injected into the disc space to promote the leakage of NP. In the TNF group (n = 5), 5 μg of TNF-α (Recombinant Rat TNF-α, R & D Systems, Minneapolis, Minnesota, United States) was placed onto the DRG. The naïve group (n = 5) was left without any surgical procedure. Post-operative pain was monitored according to the ethical permission.

Twenty-four hours after the surgery, the rats were euthanized with an overdose of pentobarbital (Alfatal, 100 mg mL^−1^, Omnidea AB, Stockholm). The exposed DRGs from the operated, and the DRGs at the similar level in naïve rats, were harvested.

### 2.2 RNA extraction and normalization

A steal bead and Tissuelyser (Qiagen AB, Sollentuna, Sweden) was used at 20Hz for 2 × 2 min to homogenize the DRGs. The supernatant was extracted with RNeasy micro Kit (Qiagen AB, Cat no 74004) according to the manufacturer's instructions. The extraction included treatment with Dnase. A spectrophotometer (Nanodrop, Thermo Fisher Scientific, Gothenburg, Sweden) was used to analyze the concentration and purity of the RNA samples. 10 ng RNA in 4 μl H_2_O-solution was used for the following steps.

### 2.3 Reverse transcription, pre-amplification and quality control

1 μl solution (5 μg/ml BSA (Thermo Fisher Scientific, Cat no B14) and 1 % Triton X (Sigma Aldrich AB, Stockholm, Sweden, Cat no T8787-50 ML) was mixed with the RNA sample and added to each well in a 96-well plate. 1.85 μl of the following hybridization mixture was added to each well (1.5 μl dNTP (10 mM) (Sigma Aldrich AB, Cat no D7295–20 × .2 M), 0.15 μl oligo-dT (100 μM) (oligoVN)* (Sigma Aldrich AB) and 0.2 μl 25xERCC spike-in (dil 1:1000) (Thermo Fisher Scientific, Cat no. 4456740). The plate was hybridized at 72 °C for 3 min and cooled down to 4 °C. The mixture was reverse transcribed by adding 8.15 μl of the following reverse transcription mix to each well (0.035 μl H2O (Thermo Fisher Scientific, Cat no 10977), 3 μl 5x superscript II first-strand buffer (Thermo Fisher Scientific, part of SuperScript II Kit, Cat no 18064014), 3 μl Betaine (5 M) (61962-50G, Sigma Aldrich AB), 0.75 μl DTT (100 mM) (Thermo Fisher Scientific, part of SuperScript II Kit, Cat no 18064014), 0.15 μl MgCl2 (1 M) (Thermo Fisher Scientific, Cat no AM9530G), 0.09 μl TSO (100 μM) [5‘-[Btn]AAGCAGTGGTATCAACGCAGAGTACATrGrG + G-3‘] (Eurogentec, Liège, Belgium), 0.375 μl RNaseOUT (40U/μl) (Thermo Fisher Scientific, Cat no 10777–019), 0.75 μl Superscript II reverse transcription (200U/μl) (Thermo Fisher Scientific, Cat no 18064–071) ad run at 42 °C for 90 min, 70 °C for 15 min and the cooled at 4 °C.

Pre-amplification was performed by adding the following mixture 7 μl H_2_O (Thermo Fisher Scientific, Cat no 10977), 25 μl 2x KAPA hiFi HotStart ready mix (Sigma-Aldrich AB, Cat no KK2602) and 0.5 μl IS PCR primer (10 μM) [5’-[Btn]AAGCAGTGGTATCAACGCAGAGT-3’] (Sigma Aldrich AB), and run with the following program 98 °C for 3 min, 18 cycles of (98 °C for 20 s, 67 °C for 15 s and 72 °C for 6 min), 72 °C for 5 min and then cooled at 4 °C.

Eight μl of the pre-amplified material was diluted with 232 μl TE-buffer (Thermo Fisher Scientific, Cat no AM9858) and frozen at −20 °C. The quality of the pre-amplified material was analyzed with gel electrophoresis using High Sensitivity DNA Analysis Kit (Bioanalyzer, Agilent Technologies, Santa Clara, CA, USA).

### 2.4 qPCR

For the qPCR reaction, 1 μl of the pre-amplified material was mixed was added to validated pre-spotted primer plates (Neuronal Ion Channels (SAB Target List) R384 (Bio-Rad Laboratories In, Hercules, CA, USA, Cat no 10047162) together with 5 μl SsoAdvanced™ Universal SYBR® Green Supermix (Bio-Rad Laboratories Inc) and 4 μl Rnase Free H_2_O (Thermo Fisher Scientific, Cat no 10977) and run in duplicates on a CFX 384 (Bio-Rad Laboratories Inc) with the following program: 95 °C for 2 min, 40 cycles of (95 °C for 5 s and 62 °C for 30 s) followed by melting curve: 95 °C for 5 s and 65 °C for 5 s.

### 2.5 Data analysis

Before reference gene screening, any data showing signs of primer dimers or inhibition were removed. The data was imported to the GenEx software (MultiD), an average of qPCR replicates was performed as well as a grouping of the different treatments. Any Cq-values exceeding 35 or containing more than 25 % missing data were excluded. Missing data were substituted with an average Cq-value from the corresponding group. A reference gene screening was made using the GeNorm and NormFind softwares. The data was normalized against the most suitable reference genes, relative expressions were calculated against the maximum, the data was log2 transformed, and genes were chosen for analysis. Finally, descriptive statistics and PCA plots were made.

## 3 Results

For the analyzes, Clcn7 and Hsp90ab1 were chosen as reference genes and were used for normalizing the dataset.

**Fig. 1:**
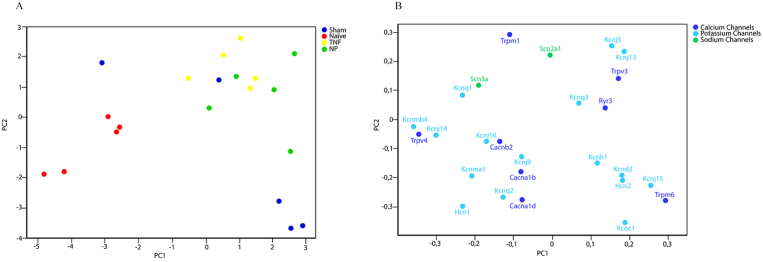
PCA plot of the expression levels of neuronal ion channel genes in dorsal root ganglion from non-operated rats (naïve) and rats that had surgery without disc puncture (sham) and rats that had surgery with disc puncture and treatment with TNF (TNF) or nucleus pulposus (NP). A) PCA plot illustrating that the naïve group (red dots) separates from the three groups that had surgery. Each dot represents one rat. B) Gene load illustrating how the different genes influences each data point in A.

**Fig. 2:**
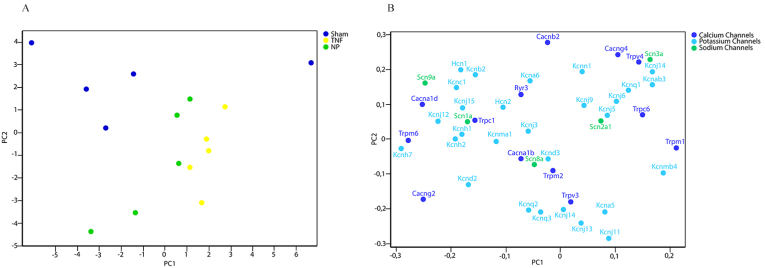
PCA plot of the expression levels of neuronal ion channel genes in dorsal root ganglion from rats that had sham surgery and rats that had surgery and application of TNF or nucleus pulposus (NP). A) PCA plot illustrating that the TNF- and NP-treated groups are commonly separated from the sham operated group. Each dot represents one rat. B) Gene load illustrating how the different genes influences each data point in A.

**Fig. 3:**
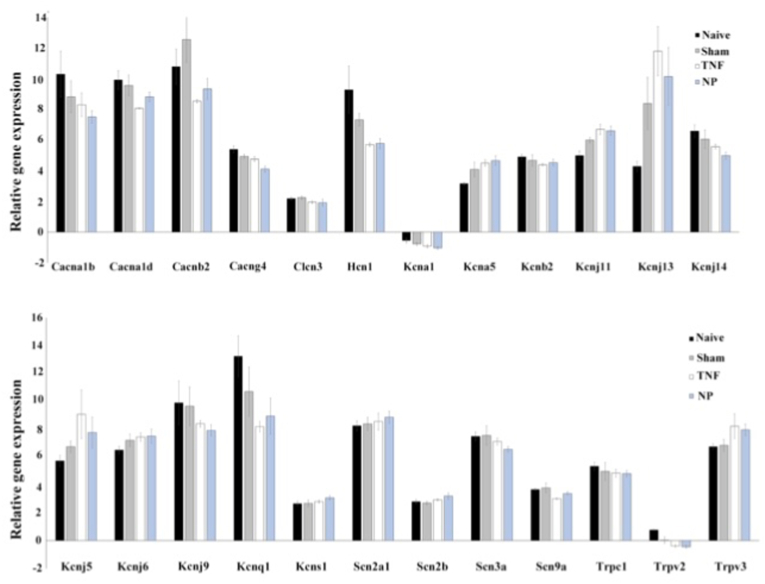
Relative expression levels of neuronal ion channel genes in dorsal root ganglion from non-operated rats (naïve) and rats that had surgery without disc puncture (sham), and rats that had surgery with disc puncture and treatment with TNF (TNF) or nucleus pulposus (NP). n = 5 for each group.

Out of the 90 genes analyzed, results were received from 84 genes after removal of insufficient data. A dynamic principal component analysis (PCA) plot was performed of the gene expression levels of the ion channels in the DRG for all samples revealing that the naïve group (red) were separated from all groups that had surgery (Sham, NP and TNF), Fig. 1. The three groups that underwent surgery were further compared in a PCA plot showing that the animals treated with TNF or NP were grouped separate from the sham operated animals, Fig. 2. When analyzing expression of the genes individually, even though not statistically significant due to a limited number of samples, the expression levels in the DRG of 24 genes showed a trend that the TNF-, and NP-treated groups commonly differed from the naïve and sham operated groups, Fig. 3. The gene expression was commonly either higher or lower in the TNF, - and NP-treated groups compared with the naïve and sham-operated groups.

## 4 Discussion

Changes in ion-channels in the rat DRG were studied after application of autologous nucleus pulposus or recombinant TNF and in sham operated rats. Naïve (non-operated) rats served as controls. It was evident that there were changes in the gene expression of the ion channels studied in the operated rats as compared to the naïve rats. It was also evident that the changes in rats with nucleus pulposus and TNF application were separated from the sham operated rats and that the changes induced seemed to be similar between nucleus pulposus and TNF application.

During recent years, there has developed an understanding that sciatic pain is a response of mechanical deformation of sensitized nerve tissue^1^^,^^2^ However, the pathophysiologic mechanisms behind such sensitization are poorly known, but it has been suggested that changes in ion channels in the membranes of the exposed nervous tissue may be involved. Indeed, a previous study indicated that local application of autologous nucleus pulposus onto DRG's in the rat might induces changes in voltage gated sodium channels.^4^

The VGSC Scn9a has been shown to have upregulated gene expression in inflamed DRG in combination with nerve compression^8^^,^^9^ and has been shown to be closely connected to pain disorders and nerve hyperexcitability.^10^^,^^11^ In this study, we instead found a downregulation of Scn9a in the TNF-group compared to both the naïve group (p = 0.009) and the NP group (p = 0.028) but no significant change compared to the sham operated group. In both studies by Mukai et al. and Huang et al. the extraction of the DRG, after intervention, was not done until after 7 days^9^ and 14 days^8^^,^^9^ compared to the 24 h until extraction that were used in this study. This indicate that the expression of Scn9a take longer time than 24 h to reach its endpoint and continues to be modulated over a longer period of time.

Other VGSC that has been targeted for evaluation, and shown upregulation has been acid-sensing ion channel 3^12^ and Scn10a.^8^ In a study from 2017 regarding nociceptor excitability associated with chronic inflammation on transgenic mice that constituently expressed TNF by Fischer et al.,^13^ they found that a significant upregulation of Scn10a and Scn11a occurred. In our study, no significant difference was found in these genes between the TNF-group and the naïve one though a significant upregulation could be seen in Scn11a between both the sham and the naïve group (p = 0.047) and the NP group compared with the naïve group (p = 0.047).

From the overall gene expression profiling we found, as expected, that all rats that had surgery grouped together separated from the non-operated naïve group, Fig. 2. Interestingly, when comparing the groups that had surgery, it turned out that the groups treated with either TNF or NP jointly differed from the sham operated group, Fig. 3. Analyzing the expression of the different genes separately, the TNF and the NP treated groups had a very similar pattern and were commonly different from the naïve and sham operated groups for 24 ion channel genes. These includes calcium channels (Cacna1b, Cacna1d, Cacnb2m Cacng4, Clcn3, Trpc1, Trpv2, Trpv3), potassium channels (Hcn1, Kcna1, Kcna5, Kcnb2, Kcnj5, Kcnj6, Kcnj9, Kcnj11, Kcnj13, Kcnj14, Kcnq1, Kcns1) and sodium channels (Scn2a1, Scn2b, Scn3a, Scn9a). Since ion channel changes following NP and TNF application have a similar, almost identical, role in gene expression of DRG, one might consider that TNF actually is the mediator of the nucleus pulposus-induced changes.

One must, however, consider that alterations in gene expression levels for any ion channel do not necessarily mean an automatic alteration in protein level synthesis. There are many pathways of posttranslational and posttranscriptional correction of protein synthesis, which does not always lead to synthesis of ion channels and assembling of a fully functional ion channel.

Additional studies on protein level are needed to analyze cellular effects of TNF or NP on alteration of ion channel expression in the DRG. However, the study indicates that there are changes in ion-channel expression induced following surgery and that application of TNF or NP induces similar changes and that they are separated from the changes induced by sham surgery. Since the changes induced by TNF and NP are similar, one might also suspect that the NP-induced changes are induced by TNF. However, such mechanism needs further investigation.

## Ethical statement

All animal experiments were performed after prior approval from the local ethics committee for animal studies at the administrative court of appeals in Gothenburg, Sweden, and in accordance with EU Directive 2010/63/EU for animal experiments.

## Funding statement

The institution of the corresponding author (KO) has received funding from AFA Insurance, Stockholm, Sweden.

## Patients consent

Not applicable – no patients were involved in this work.

## CRediT authorship contribution statement

**Joakim Håkansson:** Conceptualization, Formal analysis, Investigation, Methodology, Visualization, Writing – original draft. **Oskar Juhlin:** Conceptualization, Formal analysis, Methodology, Writing – review & editing. **Armen Hovannisyan:** Conceptualization, Formal analysis, Investigation, Methodology, Visualization, Writing – original draft. **Jennifer Rosendahl:** Formal analysis, Methodology, Visualization, Writing – review & editing. **Yalda Bogestål:** Conceptualization, Formal analysis, Investigation, Methodology, Writing – original draft. **Kjell Olmarker:** Conceptualization, Funding acquisition, Investigation, Methodology, Supervision, Writing – original draft.

## Declaration of competing interest

No benefits in any form have been or will be received from a commercial party related directly or indirectly to the subject of this manuscript.
